# Early developmental carry‐over effects on exploratory behaviour and DNA methylation in wild great tits (*Parus major*)

**DOI:** 10.1111/eva.13664

**Published:** 2024-03-13

**Authors:** Bernice Sepers, Koen J. F. Verhoeven, Kees van Oers

**Affiliations:** ^1^ Department of Animal Ecology Netherlands Institute of Ecology (NIOO‐KNAW) Wageningen The Netherlands; ^2^ Behavioural Ecology Group Wageningen University & Research (WUR) Wageningen The Netherlands; ^3^ Department of Animal Behaviour Bielefeld University Bielefeld Germany; ^4^ Department of Terrestrial Ecology Netherlands Institute of Ecology (NIOO‐KNAW) Wageningen The Netherlands

**Keywords:** animal personality, brood size, catch‐up growth, development, epigenetics

## Abstract

Adverse, postnatal conditions experienced during development are known to induce lingering effects on morphology, behaviour, reproduction and survival. Despite the importance of early developmental stress for shaping the adult phenotype, it is largely unknown which molecular mechanisms allow for the induction and maintenance of such phenotypic effects once the early environmental conditions are released. Here we aimed to investigate whether lasting early developmental phenotypic changes are associated with post‐developmental DNA methylation changes. We used a cross‐foster and brood size experiment in great tit (*Parus major*) nestlings, which induced post‐fledging effects on biometric measures and exploratory behaviour, a validated personality trait. We investigated whether these post‐fledging effects are associated with DNA methylation levels of CpG sites in erythrocyte DNA. Individuals raised in enlarged broods caught up on their developmental delay after reaching independence and became more explorative as days since fledging passed, while the exploratory scores of individuals that were raised in reduced broods remained stable. Although we previously found that brood enlargement hardly affected the pre‐fledging methylation levels, we found 420 CpG sites that were differentially methylated between fledged individuals that were raised in small versus large sized broods. A considerable number of the affected CpG sites were located in or near genes involved in metabolism, growth, behaviour and cognition. Since the biological functions of these genes line up with the observed post‐fledging phenotypic effects of brood size, our results suggest that DNA methylation provides organisms the opportunity to modulate their condition once the environmental conditions allow it. In conclusion, this study shows that nutritional stress imposed by enlarged brood size during early development associates with variation in DNA methylation later in life. We propose that treatment‐associated DNA methylation differences may arise in relation to pre‐ or post‐fledging phenotypic changes, rather than that they are directly induced by the environment during early development.

## 1 INTRODUCTION

Adverse, postnatal developmental conditions are known to have instant phenotypic effects (e.g. Hane & Fox, 2006; Hunt & Simmons, 2000; Naguib et al., 2011; Saino et al., 2003). Interestingly, these phenotypic alterations are often not confined to the period in which the conditions occurred (Monaghan & Haussmann, 2015). For example, in humans, postnatal developmental stressors such as parental loss and maltreatment induce long‐lasting metabolic alterations and adult obesity (Alciati et al., 2013; Danese & Tan, 2014; Williamson et al., 2002). In laboratory rodents, reduced maternal care is known to affect offspring adiposity, hypothalamic–pituitary–adrenal reactivity and behavioural responses to stress in adulthood (e.g. Weaver et al., 2004; Yam et al., 2017). In wild animals, lingering effects of postnatal developmental stress have been found to affect the reproduction and survival (Lendvai et al., 2009; Saino et al., 2018; Vincenzi et al., 2013).

Despite the importance of early developmental stress for shaping the adult phenotype, it is largely unknown which molecular mechanisms are responsible for such delayed phenotypic effects once the stressful developmental conditions are released. Epigenetic mechanisms, such as DNA methylation, have been proposed mechanisms to explain the link between early developmental effects and adult phenotypes (Korochkin, 2006). This biochemical mechanism can modify gene expression by adding a methyl group (–CH_3_) to a DNA nucleotide. In vertebrates, DNA methylation is predominately present at cytosines in a CG dinucleotide context (i.e. CpG site) (Bernstein et al., 2007; Bird, 2002) where, in a promoter context, it generally decreases gene expression (Weaver et al., 2004) by impeding the binding of the transcription machinery (Yin et al., 2017). DNA methylation and other epigenetic mechanisms are different from other mechanisms that modulate gene expression, such as transcription factors, as epigenetic marks can be maintained during DNA replication. Therefore, epigenetic marks and the effects on gene expression can be stably inherited from cell to cell throughout an individual's lifetime (Roth et al., 2009; St‐Cyr & McGowan, 2015). As DNA methylation patterns are very dynamic during postnatal development (Jaenisch & Bird, 2003; Watson et al., 2019) and can be influenced by environmental variation (Richards, 2006), DNA methylation is often seen as a candidate mechanism to mediate the influence of developmental conditions on phenotypic traits.

So‐called carry‐over effects on DNA methylation from postnatal stress (Hanson & Gluckman, 2014) have been found in a variety of organisms ranging from humans (McGowan et al., 2009) and laboratory rodents (St‐Cyr & McGowan, 2015; Weaver et al., 2004) to fish (Metzger & Schulte, 2017) and birds (Jimeno et al., 2019). Several findings from wild populations suggest that carry‐over epigenetic changes also occur in animals in their natural habitat (Laubach et al., 2019; Rubenstein et al., 2016). However, in ecologically relevant settings, it is a challenge to disentangle the observed postnatal effects from genetic variation or from prenatal conditions.

This challenge can be overcome by studying carry‐over effects in altricial birds. As altricial birds are confined to one place (the nest) during their early development, it is relatively straightforward to control and manipulate the conditions they experience. Furthermore, once the nestlings fledge, they experience a major transition in environments and in the level of control over their new environment. Therefore, compared to other animal species, it is often very clear when the period of early developmental stress has ended. Despite this clear transition, the effects of early developmental stress are often apparent both before and extending until after becoming independent from the parents. Early developmental conditions have been found to affect individual birds even after reaching independence and into adulthood (de Kogel, 1997; Krause et al., 2009; Merilä & Svensson, 1997; Nettle et al., 2013; Ohlsson & Smith, 2001; Richner, 1992; Saino et al., 2018; Tschirren et al., 2009). Interestingly, compensatory effects might appear throughout an individual's lifetime. Once the parental control over the environment has been released, studies reported compensation in terms of wing length (de Kogel, 1997), body mass (Careau et al., 2014; Criscuolo et al., 2008; Honarmand et al., 2010; Krause & Naguib, 2011; Ohlsson & Smith, 2001) and tarsus length (Honarmand et al., 2010; Ohlsson & Smith, 2001) in individuals that were reared under poor conditions.

In addition to morphological effects, experiences during early life development can also have long‐lasting effects on behavioural traits (see van Oers et al., 2023) such as exploratory behaviour, a well‐established personality trait in passerine birds (Carere et al., 2005; Krause et al., 2009; Krause & Naguib, 2011; Naguib et al., 2011). Personality describes individual differences in behavioural tendencies that are consistent across situations and time (Stamps & Groothuis, 2010a, 2010b). Since an animal's response to the environment, among others, depends on its personality (Réale et al., 2007), personality can buffer the effect of environmental changes or disturbances (Morozov et al., 2013). On the other hand, personality trait expression itself can also be affected by the environment. For example, great tits (*Parus major*) and zebra finches (*Taeniopygia guttata*) that experienced nutritional restriction during the nestling period explored more rapidly than individuals that had experienced superior nutritional conditions (Carere et al., 2005; Krause et al., 2009).

Few studies have examined the effects of the postnatal rearing environment on DNA methylation in wild nestlings (Sepers et al., 2021; Sepers, Chen, et al., 2023; Sepers, Mateman, et al., 2023; Sheldon et al., 2018; von Holdt et al., 2023), but none examined (lasting) effects in fledglings or adults. Using a cross‐fostering and brood size manipulation experiment in wild great tits, we previously showed that nestlings from enlarged and reduced broods differ in biometric and behavioural traits, but they differed in methylation level in one CpG site only. (Sepers, Mateman, et al., 2023). Here, we follow the same birds post‐fledging and evaluate their phenotypic traits and DNA methylation later in life. Two possible reasons could explain a relationship between DNA methylation and post‐fledging phenotypic effects. First, DNA methylation changes induced early in life can potentially remain throughout an individual's life. If lasting variation in DNA methylation due to early developmental effects maintains phenotypic effects, we expect to detect the same DNA methylation differences post‐fledging compared to the nestling phase. Alternatively, DNA methylation changes that occur post‐fledging may provide organisms the opportunity to change their physiology and behaviour as a response to earlier experienced environmental changes (Feil & Fraga, 2012; Jablonka & Lamb, 2007). Thus, post‐fledging variation in DNA methylation can be caused by variation in gene expression (Höglund et al., 2020), which arose as a consequence of phenotypic effects pre‐fledging, to facilitate adaptive phenotypic plasticity post‐fledging. If this is the case, we expect to detect post‐fledging changes in DNA methylation in response to the pre‐fledging phenotypic effects.

## 2 METHODS

### 2.1 Subjects, the study site and the sample size

This study was conducted from April to September 2018, using a long‐term nest box population of great tits (*P. major*) in Westerheide, a mixed wood forest near Arnhem, the Netherlands (52°01′00 N, 05°50′30 E). The birds used in this study were part of a brood size manipulation experiment as described in detail in Sepers, Chen, et al. (2023) and Sepers, Mateman, et al. (2023). In short, we conducted a partial cross‐foster design and manipulated the original brood sizes in 32 broods. We assigned broods with the same hatch date and similar brood sizes to a cross‐foster pair (hereafter CF pair) on day one or two after hatching. Within a CF pair, the nestlings were partially cross‐fostered and brood sizes were manipulated by enlarging the original brood size of one brood by three nestlings, while the original brood size of the other brood was reduced by three nestlings (Figure S1). Right before fledging (day 14 after hatching), 118 nestlings from 12 experimentally enlarged broods and 73 nestlings from 13 experimentally reduced broods were still alive. After these nestlings had fledged, they were caught with mist nets at seven different locations distributed over a 1000 × 1200 m area from June to September 2018. The fledglings were attracted to each catching location by two feeding stations filled with sunflower seeds. Catching started 4 weeks after all birds had fledged to ensure that they were nutritionally independent as parental care does usually not continue for longer than 20 days after fledging in great tits (Verhulst & Hut, 1996). In total, 23 birds from nine enlarged broods and 36 birds from 14 reduced broods (from eight complete and seven incomplete CF pairs) were recaptured.

### 2.2 Novel environment test

After catching, the birds were brought to the indoor bird facilities at the NIOO‐KNAW, where they were weighed and individually housed in standard housing cages (0.9 × 0.4 × 0.5 m). Birds were provided with ad libitum access to sunflower seeds, egg food, mealworms (*Tenebrio molitor*) and water. The next morning, the birds were individually tested for exploration behaviour using a novel environment test (Dingemanse et al., 2002) in a novel environment room (4.0 × 2.4 × 2.3 m), containing five artificial trees and sliding doors connecting each cage to the novel environment room. The birds were stimulated to enter the room without handling, by darkening the cage with a towel and opening the sliding door to the lightened novel environment room. After a bird entered the room, all hops and flights within and between trees, walls, door, floor and ceiling were recorded for 2 min. An individual exploration score was calculated as the sum of all movements corrected for date (Dingemanse et al., 2002). All birds were tested between 08:00 and 13:00 h. After the novel environment test, the birds were weighed (using a digital scale, ±0.01 g) and the length of their third primary (P3, counting from the outside of the wing; ruler, ±0.5 mm) was measured. In addition, a blood sample (approximately 20 μL) was taken from the brachial vein. Half of the sample was stored in 1 mL of cell lysis buffer (Gentra Puregene Kit, Qiagen, USA) for DNA extraction (see Section 2.4.1), while the other half was stored in 1 mL of Queen's lysis buffer for longer preservation for potential future studies (Seutin et al., 1991). Both samples were kept at room temperature until further analysis. After testing, measuring and bleeding, the birds were released close to the place of capture.

### 2.3 Statistical analysis biometry and behavioural data

An overview of all statistical analyses is provided in Table S1. To analyse the effect of the treatment on exploratory score, P3 length and post‐fledging weight on the day before the novel environment test, separate linear mixed models (LMMs) with ML estimation were used. To specifically test whether the effect of the treatment depended on time that had passed since the bird had fledged (end of treatment), we included treatment, days since fledging and their interaction as fixed effects in the model.

To test the consequences of the treatment on the post‐fledging weight gain or loss (hereafter ∆ weight), compared to the pre‐fledging weight on day 14, an LMM with ML estimation was fitted with ∆ weight as the dependent variable. Since we previously found that the effect of the treatment on weight on day 14 after hatching changed non‐linearly over hatch date (Sepers, Mateman, et al., 2023), we included treatment, hatch date, hatch date squared and the interactions between treatment and hatch date and treatment and hatch date squared as fixed effects. To test whether the effect of the treatment depended on time that had passed since the bird had fledged, days since fledging and the interaction between treatment and days since fledging were included as fixed effects.

In the above‐described models, CF pair was not included as a fixed effect since we were not able to test individuals from both broods for most CF pairs. Brood of origin and brood of rearing nested within CF pair were included as random effects. Final models were selected following a backwards elimination procedure by progressively deleting non‐significant fixed terms (*p* < 0.05), starting with the interactions. The minimal adequate model always included treatment. All analyses were done using the packages *lme4* v1.1.27.1 and *lmerTest* v3.1.3. Post‐hoc comparisons and estimates of slopes were performed with the *emtrends* function in the package *emmeans* v1.7.2 to assess the statistical significance of slopes and the estimate of the trend for each treatment. The *p*‐values were provided by a type III analysis of variance via Satterthwaite's degrees of freedom method and corrected for multiple testing with a Bonferroni correction.

To be able to determine whether the effects of days since fledging, date of testing and hatch date were independent, the correlations between days since fledging and hatch date and between days since fledging and date of testing were tested using Spearman's rank‐order correlations.

### 2.4 DNA methylation analysis: sample selection and processing

From the 59 recaptured fledglings, we selected 28 samples from 14 reduced broods and all 23 samples from nine enlarged broods (from eight complete CF pairs and seven incomplete CF pairs). We selected siblings that were raised in different nest boxes (i.e. treatments), while keeping the number of samples from moved and unmoved individuals as equal as possible. The 51 post‐fledging samples were pooled with 21 other samples from a different experiment to generate two sequencing libraries, each containing 36 individual samples. We made sure that each sequencing library contained samples from both treatments to prevent that a possible library or lane effect is confounded with treatment effects.

#### 2.4.1 epiGBS2 library preparation and sequencing

Individual DNA methylation was assessed at the CpG site (hereafter CpG) level using the reduced‐representation bisulfite sequencing method epiGBS2 (Gawehns et al., 2022). EpiGBS2 library preparations were conducted at the NIOO‐KNAW following the laboratory protocol described in the supporting information of Sepers, Mateman, et al. (2023). In short, 800 ng DNA per sample was extracted using the FavorPrep 96‐Well Genomic DNA Extraction Kit (Favorgen) and digested with the restriction enzyme MspI (NEB) to cut the DNA at C^CGG motif sites. Subsequently, the fragments were ligated to a unique barcode combination for each sample and the fragments from 36 samples were pooled (i.e. multiplexed). To be able to differentiate methylated cytosines from unmethylated cytosines, unmethylated cytosines were converted to thymines using sodium bisulfite. Sequencing was performed by Novogene (Novogene (HK) Company Limited, Hong Kong) on an Illumina HiSeq X (150 bp from paired‐end, directional reads). PhiX DNA was spiked‐in (12%).

### 2.5 Bioinformatics and statistical analysis: DNA methylation

We analysed the epiGBS2 output according to the recommendations in Laine et al. (2022), which include removal of overlap between read pairs, removal of sites with biased DNA methylation estimation, coverage filtering, percentile filtering and correction for overdispersion. The analyses are described in the text below.

#### 2.5.1 Bioinformatics analysis

The bioinformatics analysis was done in the same way as described in detail in Sepers, Chen, et al. (2023) and Sepers, Mateman, et al. (2023). Using the epiGBS2 bioinformatics pipeline (Gawehns et al., 2022), PCR clones were removed, raw reads were demultiplexed and reads were strand annotated as either reads originating from the top DNA strand (i.e. Watson reads) or reads originating from the bottom DNA strand (i.e. Crick reads). Next, to facilitate paired‐end alignment (see below), all Watson and Crick R1 reads and all Watson and Crick R2 reads were merged using a custom script in bash to create one R1 file and one R2 file per individual. Reads shorter than 20 bp and 3′ end adapter sequences were removed by discarding the Illumina sequence plus 10 bp using Cutadapt v2.10 (Martin, 2011). Subsequently, alignment of the trimmed reads to the *P. major* reference genome v1.1 (GCF_001522545.3) (Laine et al., 2016) was done in paired‐end and non‐directional mode using Bismark v0.22.3 (Krueger & Andrews, 2011) with Bowtie2 v2.3.5.1 (Langmead & Salzberg, 2012). The average mappability was 45.65% (range: 40.00%–48.30%). Methylation calling was done in CpG context only and in paired‐end mode while removing overlap between read pairs and the first four bp's using Bismark (Krueger & Andrews, 2011). Throughout the bioinformatics analysis, read quality was assessed using FastQC v0.11.8 (Andrews, 2010), FastQ screen v0.11.1 (Wingett & Andrews, 2018) and MultiQC v1.8 (Ewels et al., 2016).

#### 2.5.2 Filtering of methylation calls

After methylation calling, complementary CpG dinucleotides were merged using the R package *methylKit* v1.16.1 (Akalin et al., 2012). Using a custom R script, lowly (<10×) and highly covered CpG sites (>99.9th percentile of coverage) and CpG sites (hereafter CpGs) that were not covered in minimally 15 individuals in both treatments were discarded. Next, we calculate the mean methylation level per CpG (methylated cytosines divided by the coverage) and discarded CpGs with a high (>0.95) or low (<0.05) mean methylation level across all individuals. After filtering, 116,064 out of the 4,032,127 CpGs were used for the statistical analysis (Table S2).

#### 2.5.3 Statistical analysis: DNA methylation

To assess post‐fledging differential methylation between fledglings of different experimental groups, we fitted two generalized linear mixed models (GLMMs) with a logit link function and a binomial error structure (negative binomial) for every CpG. The dependent variable was modelled as the fraction of the number of methylated cytosines over the coverage using the *cbind* function. In the first model (i.e. null model), we included only treatment as a fixed effect. To test whether treatment‐dependent methylation differences occurred as a function of the time after the birds fledged, we ran a second model in which we included treatment, days since fledging and the interaction between the two as fixed effects. The two models described above contained brood of origin and brood of rearing, both nested within CF pair, as random effects. Sex was not included in any of the statistical models. Although male great tit nestlings might have a competitive advantage in suboptimal conditions, which can result in sex‐dependent effects of enlarged brood size (Dhondt, 1970; Drent, 1984; Lessells et al., 1996; Nicolaus et al., 2009; Oddie, 2000; Smith et al., 1989), brood size affected pre‐fledging traits in the individuals in this study regardless of sex (Sepers, Mateman, et al., 2023). Furthermore, we did not find any CpG sites that were significantly differentially methylated between the sexes in the pre‐fledging samples (Sepers, Chen, et al., 2023).

We determined the significance of the interaction between treatment and days since fledging with a likelihood ratio test by comparing the second with the first model using the R packages *lme4* v1.1.27.1 and *lmerTest* v3.1.3 and the *anova* function in R. We used a false discovery rate (FDR) (Benjamini & Hochberg, 1995) corrected *p*‐value threshold of 0.05 based on 116,064 analysed CpGs. CpGs for which the effect of the treatment depended on days since fledging are referred to as days‐dependent DMS. Models of sites that received error warnings were omitted, except when those were singularity warnings only. We corrected for overdispersion by removing CpGs that fell out of the 95% highest density interval of the distribution of the dispersion statistic for the second GLMM (Zuur et al., 2013), using the R package *HDInterval* v0.2.2.

In the case of significant interactions of treatment with days since fledging, post hoc comparisons and estimates of slopes were performed with the *emtrends* function in the package *emmeans* v1.7.2. *p*‐Values were corrected for multiple testing using an FDR of 0.05 (Benjamini & Hochberg, 1995). Based on the *p*‐values and the trends, CpGs were associated with one of the six categories corresponding to different types of interactions (Figure S2). An overview of how many CpGs were left after each statistical filtering step is provided in Table S3.

#### 2.5.4 Gene annotation

All CpGs were annotated to genomic regions: transcription start site (TSS) region (300 bp upstream – 50 bp downstream of the annotated transcription start site), promoter region (2000 bp upstream – 200 bp downstream of the annotated transcription start site), gene body (introns and exons), five prime untranslated region (5′UTR), three prime untranslated region (3′UTR), upstream regions and downstream region (10 kbp adjacent to the gene body). If a site was associated to multiple regions, we prioritised regions in this same order as described above. If a site was associated to both upstream and downstream regions of genes, we prioritised characterised genes over functionally uncharacterised genes (hereafter: LOC genes) and selected the gene of which the distance to the gene body was shortest. Annotation was done using the *P. major* reference genome build v1.1, annotation version 102 (Laine et al., 2016), R packages *rtracklayer* v1.50.0 (Lawrence et al., 2009) and *GenomicFeatures* v1.42.3 (Lawrence et al., 2013) and custom R scripts.

All analyses were done using R v4.1.0 and Rstudio v1.4.1717.

#### 2.5.5 Gene ontology analysis

To facilitate the biological interpretation of the genes in which days‐dependent DMS were found, we used the ClueGO v2.5.8 (Bindea et al., 2009) plug‐in for Cytoscape v3.9.0 (Shannon et al., 2003) to identify enriched significantly Gene Ontology (GO) terms. The target list consisted of all genes in which a days‐dependent DMS was found. The background list consisted of all genes in which a CpG was situated in our overall dataset after filtering (so before running the models). LOC genes were excluded as there were too many LOC genes in the background list to check manually (2691 LOC genes) and we wanted to keep the comparison between the target list and the background list as unbiased as possible. The human annotations (01‐03‐2017) and the ontologies biological process, molecular function and cellular component and the Kyoto Encyclopedia of Genes and Genomes (KEGG) pathway (Kanehisa & Goto, 2000) were used (13‐05‐2021). ClueGO was run such that a GO term was selected if minimally 5% of the genes in the target list were associated with the GO term and minimally five were genes associated with the GO term. The network specificity was set to ‘medium’ ranging from the third to the tenth GO level. Furthermore, a two‐sided enrichment/depletion test and FDR correction for multiple testing were applied. GO categories with an FDR corrected *p*‐value of <0.05 were considered significantly enriched.

To verify the results obtained with ClueGO, we also used String v11.5 (Szklarczyk et al., 2021) to identify enriched GO terms and to construct protein–protein interactions. The same annotations, target list of genes and correction for multiple testing as described above were used and we applied a confidence cut‐off of 0.7, although relaxed cut off values gave the same result.

## 3 RESULTS

### 3.1 Treatment effects on the biometry and behaviour

**FIGURE 1:**
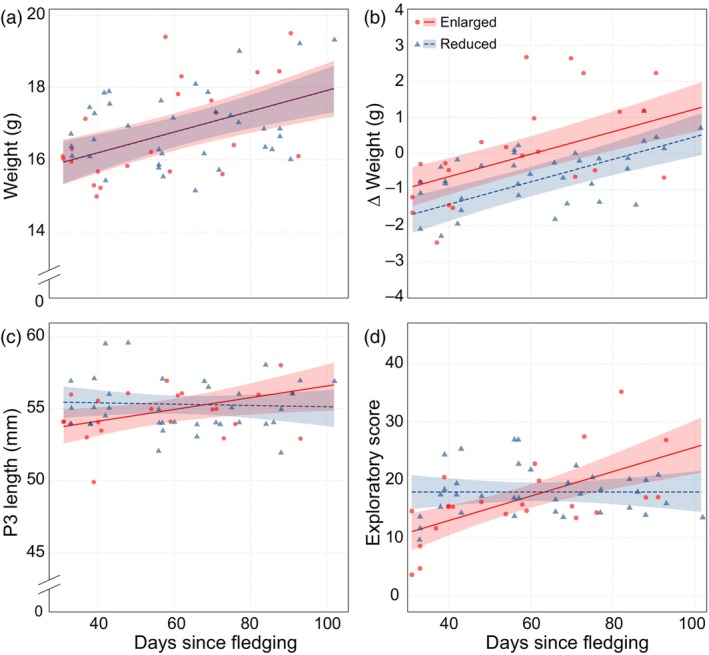
Post‐fledging weight, ∆ weight, P3 length and exploratory score. Relationship between days since fledging and (a) post‐fledging weight (g), (b) ∆ weight (g), (c) P3 length (mm) and (d) exploratory score for both treatments. ∆ Weight was calculated as post‐fledging weight minus day 14 weight. Raw data points, regression lines and 95% confidence intervals are plotted for individuals from the reduced treatment (blue) and the enlarged treatment (red).

When we caught birds several weeks after fledging, the time between fledging and recapture did affect post‐fledging weight (days since fledging, LMM: *F*_1,52.85_ = 19.69, *p* < 0.001) where post‐fledging weight increased with days since fledging (Figure 1a; Table S4). However, this was irrespective of whether individuals originated from enlarged or reduced broods (treatment, LMM: *F*_1,20.15_ = 0.0004, *p* = 0.98).

**FIGURE 2:**
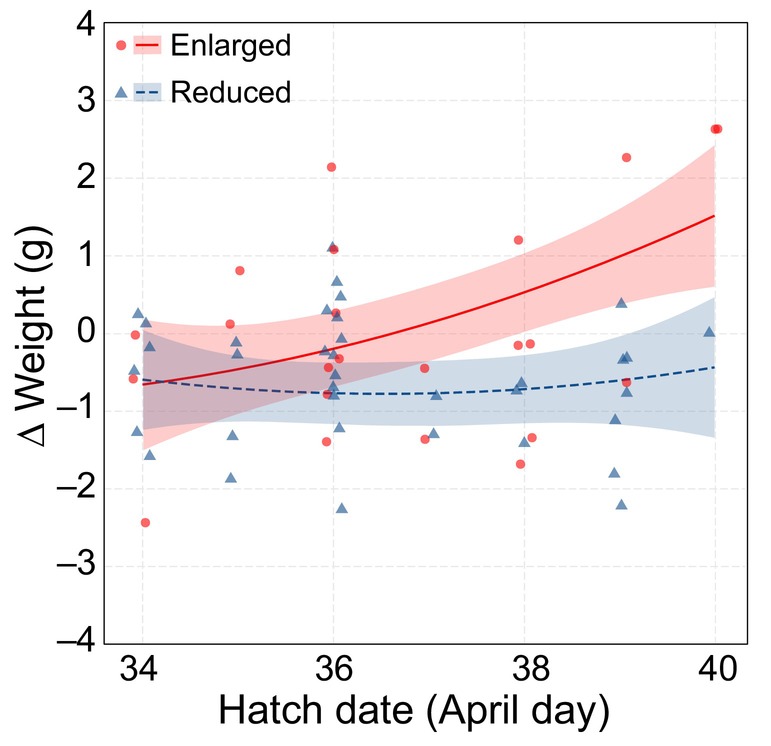
Relationship between hatch date (April day) and ∆ weight (g) for both treatments. ∆ Weight was calculated as post‐fledging weight minus day 14 weight. Raw data points, regression lines and 95% confidence intervals are plotted for individuals from the reduced treatment (blue) and the enlarged treatment (red).

This compensation (the difference between pre‐ and post‐fledging weight) did show a non‐linear season‐dependent treatment effect (treatment × hatch date^2^, LMM: *F*_1,19.45_ = 5.68, *p* = 0.03; Table S5; Figure 2). Birds that hatched from enlarged broods, progressively gained more weight if they were recaptured, when they hatched later in the season (*β* ± SE = 0.005 ± 0.002, *t*.ratio_19.4_ = 3.03, *p* = 0.01; Figure 2). In contrast, birds that were raised in reduced broods did not show such catch‐up growth (*β* ± SE = 0.00005 ± 0.001, *t*.ratio_19.4_ = 0.04, *p* = 1.00; Figure 2). Although weight gain increased when birds were captured later after fledging (days since fledging, LMM: *F*_1,50.02_ = 39.006, *p* < 0.001; Figure 1b), the treatment‐dependent weight gain was not dependent upon the days since fledging (treatment × days since fledging, LMM: *F*_1,47.03_ = 2.45, *p* = 0.12; Table S5).

Treatment effects on P3 length at catching were dependent on the time that passed between fledging and catching (treatment × days since fledging, LMM: *F*_1,42.77_ = 4.29, *p* = 0.04; Table S6). While P3 length increased with the number of days that had passed since fledging in birds from enlarged broods (*β* ± SE = 0.04 ± 0.02, *t*.ratio_33.1_ = 2.38, *p* = 0.047; Figure 1c), birds from reduced broods did not show such an effect (*β* ± SE = −0.01 ± 0.01, *t*.ratio_58.2_ = −0.42, *p* = 1.00; Figure 1c). Exploratory behaviour showed the same time‐dependent treatment effect as P3 length (treatment × days since fledging, LMM: *F*_1,57.72_ = 11.24, *p* = 0.001; Table S7). Birds from reduced broods did not vary in exploratory score depending on the time that passed between fledging and catching (*β* ± SE = 0.002 ± 0.04, *t*.ratio_59.0_ = 0.04, *p* = 1.00; Figure 1d), while the exploratory score of birds from enlarged broods increased with the number of days since fledging (*β* ± SE = 0.21 ± 0.05, *t*.ratio_54.4_ = 4.39, *p* < 0.001; Figure 1d).

No significant correlation between days since fledging and hatch date was found (Spearman's rank‐order correlation; *r*_57_ = −0.15, *p* = 0.26), indicating that the number of days between fledging and the moment an individual was recaptured is independent of an individual's hatch date. We found a positive correlation between the days since fledging and date of testing (Spearman's rank‐order correlation; *r*_57_ = 0.99, *p* = 0.22 × 10^−15^), indicating that we cannot disentangle effects of the actual time that passed since fledging from seasonal effects at catching, although we expect the latter to be less important.

### 3.2 Treatment effects on DNA methylation

**FIGURE 3:**
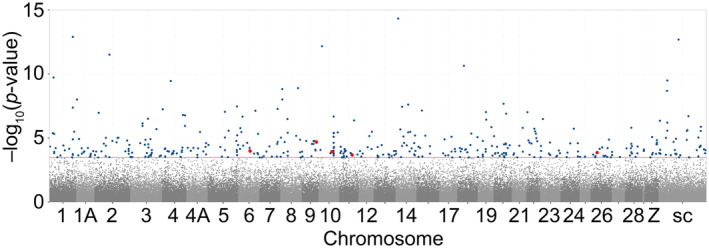
Manhattan plot showing the −log_10_(*p*)‐values corresponding to the significance of the interaction of treatment (reduced or enlarged) with days since fledging in explaining variation in DNA methylation. Each dot represents a CpG tested for a days since fledging‐dependent treatment effect (107,635 CpGs). Dark blue dots represent CpGs for which a significant days since fledging‐dependent treatment effect was found (420 CpGs). Red dots represent CpGs which are located in the TSS region of a gene and for which a significant days since fledging‐dependent treatment effect was found (five CpGs). The dotted red line marks the genome wide significance threshold. The sites are plotted against the location of the associated site within the genome. Alternating colours help to differentiate adjacently displayed chromosomes. ChrZ is a sex chromosome, all the other chromosomes are autosomes. All unplaced scaffolds are merged into the category ‘scaffolds’.

**FIGURE 4:**
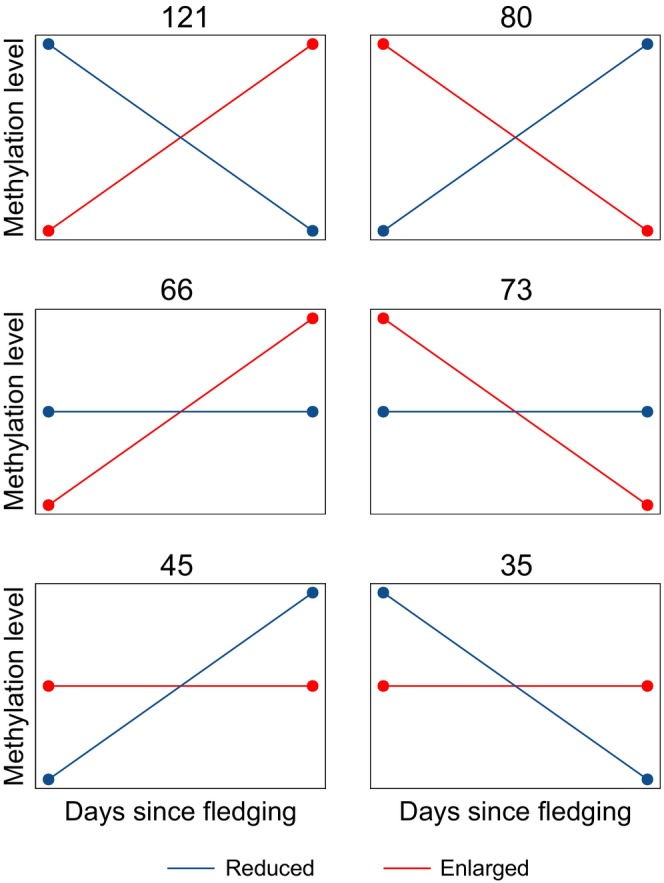
Categories corresponding to the different types of interactions between treatment (reduced or enlarged) and days since fledging in explaining variation in DNA methylation. The 420 CpGs for which this interaction was significant were divided over the six categories. The number above each plot represents the number of CpGs for which the interaction follows the pattern as depicted in the plot. Red lines represent the patterns in the enlarged treatment, dark blue lines represent the patterns in the reduced treatment. True slopes, intercepts and point of intersection may deviate slightly from the schematic representation.

We found 420 CpGs that differed between fledglings originating from reduced versus enlarged broods, where the methylation difference was associated with the days that passed between fledging and catching (420 days‐dependent DMS; Figure 3; Table S3). After performing post hoc tests, these 420 CpGs could be divided into three categories. (1) In 201 CpGs, we found contrasting patterns between fledglings from reduced and from enlarged fledglings. Of these, in 121 CpGs methylation increased with days since fledging in individuals that fledged from enlarged broods, while it decreased in individuals from reduced broods (Figure 4). In the other 80 CpGs, this pattern was reversed. (2) In 139 CpGs, methylation was stable in individuals that fledged from reduced broods, while it either increased (66 DMS) or decreased (73 DMS) with days since fledging in individuals from enlarged broods. (3) In only 80 CpGs, methylation was stable in individuals that fledged from enlarged broods, while it increased (45 DMS) or decreased (35 DMS) with days since fledging in individuals from reduced broods (Figure 4).

**FIGURE 5:**
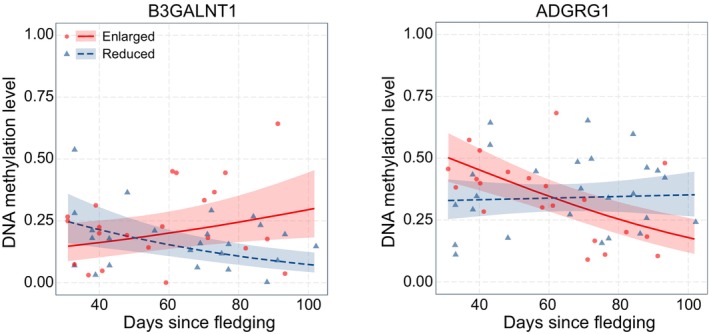
Examples of relationships between days since fledging and CpG methylation level for both treatments. Two genes with CpGs that were situated in a TSS region and showed a significant treatment effect that depended on the days that passed since fledging are displayed: B3GALNT1 and ADGRG1. Raw data points, regression lines and 95% confidence intervals are plotted for samples from the reduced treatment (blue) and the enlarged treatment (red).

Out of the 420 days‐dependent DMS, 336 could be annotated (Table S8). These sites were found in or near 282 genes, of which 237 were characterised genes and 45 were LOC genes. Out of the 336 sites, 67 were found in a promoter region of genes, of which five were situated in a TSS region of the genes heparanase 2 (*HPSE2*), SKI family transcriptional corepressor 1 (*SKOR1*), olfactomedin like 3 (*OLFML3*), beta‐1,3‐*N*‐acetylgalactosaminyltransferase 1 (*B3GALNT1*) and adhesion G protein‐coupled receptor G1 (*ADGRG1*) (Figure 5). Furthermore, 199 sites were found in a gene body and 70 sites were found upstream or downstream of genes (Table S8). A GO analysis on the 237 genes detected 96 significantly enriched GO and KEGG terms at an FDR corrected *p*‐value threshold of 0.1 (Tables SS9–S12). Our protein network analysis showed significantly more interactions than expected (*p*‐value = 0.008) (Figure S3). This analysis detected 23 significantly enriched GO terms (FDR corrected *p*‐value < 0.05), which were comparable to the ones found in the GO analysis (Tables S13 and S14). The significant terms from both analyses were mainly involved in functions concerning cell signalling, vesicle and ion channel transport, metabolism, neuronal function, mechanosensing and the development of neurons, organs and anatomical structures.

## 4 DISCUSSION

Adverse environmental conditions during postnatal development could have life‐long phenotypic consequences, possibly mediated via DNA methylation. Earlier, we found that there were strong phenotypic effects of experimental brood size, but we hardly found effects on DNA methylation (Sepers, Mateman, et al., 2023). Here, we explored this further by tracking DNA methylation several months after the experiment. As time after fledging passed, the individuals from enlarged broods caught up on their developmental delay in weight and third primary length. Furthermore, we found that fledglings from enlarged broods that were tested later, became faster explorers, while the exploratory scores of individuals from the reduced broods remained stable over time. These phenotypic effects suggest compensatory growth of birds that experienced nutrient stress during development. The post‐fledging compensatory effects were associated with DNA methylation changes that occurred post‐fledging: we found many CpGs that showed patterns of differential methylation similar to the phenotypic catch‐up effects in a time since fledging‐dependent manner.

### 4.1 Post‐fledgling biometry

The treatment effect on nestling weight we previously found (Sepers, Mateman, et al., 2023) did not last; individuals from the enlarged broods caught up on weight after fledging. We also observed a positive relationship between the change in weight and days since fledging, irrespective of the treatment. Nevertheless, individuals from the enlarged treatment did gain more weight. This indicates that individuals from larger broods needed some time after fledging to regain their body mass to meet a level comparable to individuals from the reduced broods. This compensatory growth has also been reported in several other studies (Careau et al., 2014; Criscuolo et al., 2008; Honarmand et al., 2010; Krause & Naguib, 2011; Ohlsson & Smith, 2001). In contrast to the nestling period, juveniles can actively cope with challenging situations after fledging, as they are not (solely) dependent on their parents for nutrition. We will discuss the possible ways that the fledglings could have coped with early nutritional stress in the Sections 4.2, 4.4 and 4.5.

We observed a positive relationship between the day of catching and the length of the third primary in individuals from enlarged broods, but not in individuals from the reduced broods. This is supported by a previous study, in which great tit nestlings from enlarged broods had shorter wing lengths compared to nestling from unmanipulated broods (Martyka et al., 2021). As great tits do moult their primaries during the post‐breeding moult (Jenni & Winkler, 2020), it is impossible that the observed effect is related to moulting. In great tits, wing growth slows down towards the end of the nestling period, but still continues after fledging (Orell, 1983). On day 15 after hatching, wing length is only about 2/3 of the full‐grown length of the full grown values of 70–78 mm in great tits (Gosler, 1993). Therefore, this suggests that the nestlings from the enlarged broods were developmentally delayed compared to nestlings from the reduced broods and that they caught up after fledging.

### 4.2 Exploratory behaviour

We found that exploratory scores of individuals from the enlarged treatment increased the later we caught them since they had fledged, while the exploratory scores of individuals from the reduced broods remained stable over time. Increased levels of exploratory behaviour as a consequence of poorer nutritional conditions were previously shown in great tits (Carere et al., 2005; Serrano‐Davies et al., 2023) and zebra finches (Krause et al., 2009). The changes in exploratory behaviour in individuals from enlarged broods may be interpreted as an increased level of phenotypic plasticity due to early‐life developmental constraints that becomes apparent only later in life. There are two alternative explanations for this. First, an increase in exploratory behaviour might be a consequence of the delayed development during the nestling stage and might have facilitated catch‐up growth in fledglings from enlarged broods as a highly explorative phenotype might aid in acquiring resources more quickly and in meeting the necessary amounts of ingested calories needed. This is supported by our finding that the difference in weight between individuals from enlarged and reduced broods that was present before fledging had faded by the time they were recaptured.

Alternatively, increasing levels of exploratory behaviour over time might also be a consequence of catch‐up growth instead of a facilitator. Shortly after becoming independent from their parents, individuals from the enlarged broods were slow compared to individuals from the reduced broods, while this was reversed in older individuals. It might be that young individuals from enlarged broods did not have the resources to express highly explorative behaviour right away, due to physiological constraints. When they further developed and their condition improved (after gaining weight and growing their wings), they might have been able to express or afford high explorative behaviour (Luttbeg & Sih, 2010). In the current study, we cannot distinguish between these alternative explanations. A negative relationship between basal metabolic rate and exploration behaviour has been observed in great tits (Bouwhuis et al., 2014), but only in females and a relationship between calorie intake and exploratory behaviour could not be confirmed (Serrano‐Davies et al., 2017). However, these great tits were temporally housed in captivity with ad libitum food and they were older than the birds in our study. A relationship between calorie intake and personality might only appear in a certain critical period during development.

### 4.3 Post‐fledging DNA methylation

In contrast to pre‐fledging DNA methylation (Sepers, Mateman, et al., 2023), we found effects of experimental brood size on post‐fledging DNA methylation in many sites and these effects depended on the time between fledging and catching. Since the time‐dependent treatment effects on DNA methylation mirror observations in some phenotypes, we expect these effects to reflected changes in condition over time rather than chronological age. However, just like with exploratory behaviour, we do not know whether these DNA methylation changes were a facilitator of post‐fledging phenotypic changes or whether DNA methylation changes are a consequence of processes related to catch‐up growth.

### 4.4 DNA methylation and metabolism and growth

Many DMS were found in genes related to metabolism, development and growth (Table S15). The functions of these genes were supported by enriched GO terms such as anatomical structure morphogenesis and development, cellular developmental process, growth factor activity, aminoglycan metabolic process and biosynthetic process, glycosaminoglycan metabolic process. This might indicate that differential expression of these genes facilitates retention of nutrients and minerals and/or a more efficient (Hales & Barker, 1992) or plastic metabolism via changes in DNA methylation in the individuals from enlarged broods, which might have allowed them to catch‐up in terms of growth after fledging. This suggests that the consequences of early life conditions might be mediated by adjusting post‐fledging metabolic efficiency via DNA methylation.

### 4.5 DNA methylation and behaviour and cognition

Besides a more efficient metabolism, animals can also meet increased energetic demands and facilitate rapid growth and development via the adjustment of their foraging strategy. This is supported by our findings, as we found an effect of experimental brood size on exploratory behaviour, which has also been linked to variation in foraging strategies as it is correlated with risk‐taking (Quinn et al., 2012; van Oers et al., 2004, 2005), formation of foraging routines (Verbeek et al., 1994), competitive foraging ability (Cole & Quinn, 2012) and dietary preference (Serrano‐Davies et al., 2017). Recently, we also found that great tits that were raised in low‐quality habitat differed in their exploratory behaviour and their winter foraging behaviour (Serrano‐Davies et al., 2023). In addition, we found three day‐dependent DMS in the gene norrin cystine knot growth factor (*NPD*), which expression is affected by diet (Nyunt et al., 2019). This suggests that there were differences in foraging strategies over time and between fledglings from the enlarged and reduced broods.

The functions of several affected genes also support the found effect on exploratory behaviour, as they are linked to neuronal, cognitive and behavioural changes (Table S16). Several DMS were found in or near *TPH2*, a gene involved in the biosynthesis of serotonin and genes from the *SLC* group. Polymorphisms and DNA methylation in another member of the *SLC* group (*SLC6A4* or *SERT*), which protein transports serotonin, have been linked to adverse early life conditions and stress responses in both humans and animals models (see Non et al., 2016; Parade et al., 2021). Serotonin might be important for the expression of exploratory behaviour, as a *SERT* polymorphism has been associated to exploratory behaviour in the great tit and methylation levels were found to be lower in forest‐dwelling compared with urban great tits (Riyahi et al., 2015). As urban birds are known to be faster explorers than forest‐dwelling birds (Charmantier et al., 2017; Riyahi et al., 2017), this could indicate a role for DNA methylation in the expression exploratory behaviour via mediating *SERT* expression. However, the found association between DNA methylation and exploratory behaviour was not straightforward (Riyahi et al., 2015). Previously, we investigated the differences in DNA methylation between lines artificially selected for fast and slow exploratory behaviour. Although we found differential methylation in the promoter region of one candidate gene, *DRD4*, between individuals from the fast and slow selection lines (Verhulst et al., 2016), we did not detect stably inherited epigenetic marks in a genome‐wide analysis (van Oers et al., 2020). This may suggest that behaviour‐related DNA methylation is more important for plastic responses to the environment than for heritable trait differences. Overall, our results suggest that nutritional stress mediates DNA methylation patterns of genes involved in the differential regulation of post‐fledging nervous system development and behaviour between fledglings from enlarged and reduced broods.

### 4.6 Possible sampling biases and future directions

In the great tit, exploratory behaviour has been associated to natal dispersal (Dingemanse et al., 2003). As there were time‐dependent differences between individuals from the enlarged and reduced broods in exploratory behaviour, this raises the question whether the great tits from the two treatments differed in distance or probability to disperse. This could have resulted in a treatment‐induced sampling bias as the individuals from one treatment were less likely to be captured, depending on time after fledging. Great tits can disperse shortly after nutritional independence, but the distance depends on habitat quality (Drent, 1984). Given the size of Westerheide and the provided sunflower seeds at seven different locations, we do expect that most birds stayed in the area. Furthermore, great tit dispersal has not been found to be affected by manipulated brood size (Smith et al., 1989). Thus, even if there was a difference in dispersal probability or distance between the two treatments, we do not expect that this resulted in a sampling bias.

However, such a bias might also arise due to difference in survival between the treatments. Fledging survival (Smith et al., 1989) and recruitment probability (Lendvai et al., 2009) have been found to be lower in experimentally enlarged broods. In our study, recruitment probability was almost twice as high in the reduced group than in the enlarged group (30.51% vs. 16.43%). Therefore, we cannot rule out that the patterns we found were affected by treatment‐dependent selective disappearance. On the other hand, differences in survival could also be mediated by variation in DNA methylation, which provides opportunities for future studies on the link between survival and DNA methylation. Moreover, if individuals of lower condition in the enlarged group had lower chances to survive, our effect is actually an underestimation of the actual effect, since they needed to catch‐up more compared to the surviving individuals.

Finally, validation studies are needed to elucidate the biological significance of the days‐dependent DMS. Since CpG methylation within regulatory regions (promoter and TSS regions) is known to affect gene expression in great tits (Laine et al., 2016), we expect genes associated with DMS within the regulatory region to be good candidates for explaining carry‐over effects of the early‐life environment on phenotypic traits. However, experiments are needed to assess causal effects of changes in DNA methylation of these genes on gene expression and to validate the role of DNA methylation in mediating carry‐over effects of early environmental conditions on phenotypic traits. Good candidate genes for future studies are *SLC25A6*, *SLC4A9*, *SKOR1*, *GPR182*, *B3GALNT1* and *ADGRG1* for effects on growth and *SLC25A6*, *SLC4A9*, *NEDD4L*, *RNF24*, *ADGR1* and *SIRPB1*‐like for effects on exploratory behaviour.

## 5 CONCLUSIONS

This study shows that nutritional stress caused by experimentally enlarging broods has, apart from short‐term consequences on body mass, more delayed consequences on DNA methylation. Since we found an effect of early developmental stress on post‐fledging DNA methylation in the present study, but hardly on DNA methylation pre‐fledging (Sepers, Mateman, et al., 2023), we hypothesise that DNA methylation differences later in life have arisen as a consequence of the effects on gene expression (Höglund et al., 2020) and phenotypic changes due to early life stress. The post‐fledging methylation changes may provide organisms with the opportunity to adaptively modulate their wing size and exploratory behaviour once environmental conditions allow it. This then leads to the so‐called carry‐over phenotypic effects, with possible long‐lasting effects for an individual. Given that effects on DNA methylation were present in functionally relevant genes with effects on behaviour and growth, our results support the hypothesis that plasticity of phenotypic traits may be mediated via DNA methylation. Nevertheless, our study cannot make causal inferences and we cannot rule out the opposite, that DNA methylation changes are a consequence of post‐fledging changes in phenotypic traits. Together, our results suggest that DNA methylation variation plays a less important role during early development than previously assumed, but under control of gene expression might affect carry‐over effects later in life.

## FUNDING INFORMATION

This work was primarily supported by an NWO‐ALW open competition grant (ALWOP.314) to Kees van Oers. Bernice Sepers was funded by a Bielefeld Young Researchers' Fund scholarship from Bielefeld University during part of the work.

## CONFLICT OF INTEREST STATEMENT

The authors declare that there are no conflicts of interest.

## Supporting information


Appendix S1

## Data Availability

The multiplexed epiGBS2 reads are publicly available on NCBI under BioProject ID PRJNA208335 under the SRA accessions SRX21758799 and SRX21758800. The epiGBS2 bioinformatics pipeline can be accessed on Github (https://github.com/nioo‐knaw/epiGBS2). All other scripts and the biometric and behavioural data are publicly available on Dryad (https://doi.org/10.5061/dryad.hqbzkh1pf).
