# Supplementary material for: Early developmental carry‐over effects on exploratory behaviour and DNA methylation in wild great tits (Parus major)
Source: Evol Appl. 2024 Mar 13;17(3):e13664. doi: 10.1111/eva.13664 (PMC10937296; doi:10.1111/eva.13664)
Supplement: Supplementary file 1 — Appendix S1 [file EVA-17-e13664-s001.docx]

**Supporting Information for:**

Early developmental carry-over effects on exploratory behaviour and DNA methylation in wild great tits (*Parus* *major*)

Bernice Sepers, Koen J.F. Verhoeven, Kees van Oers


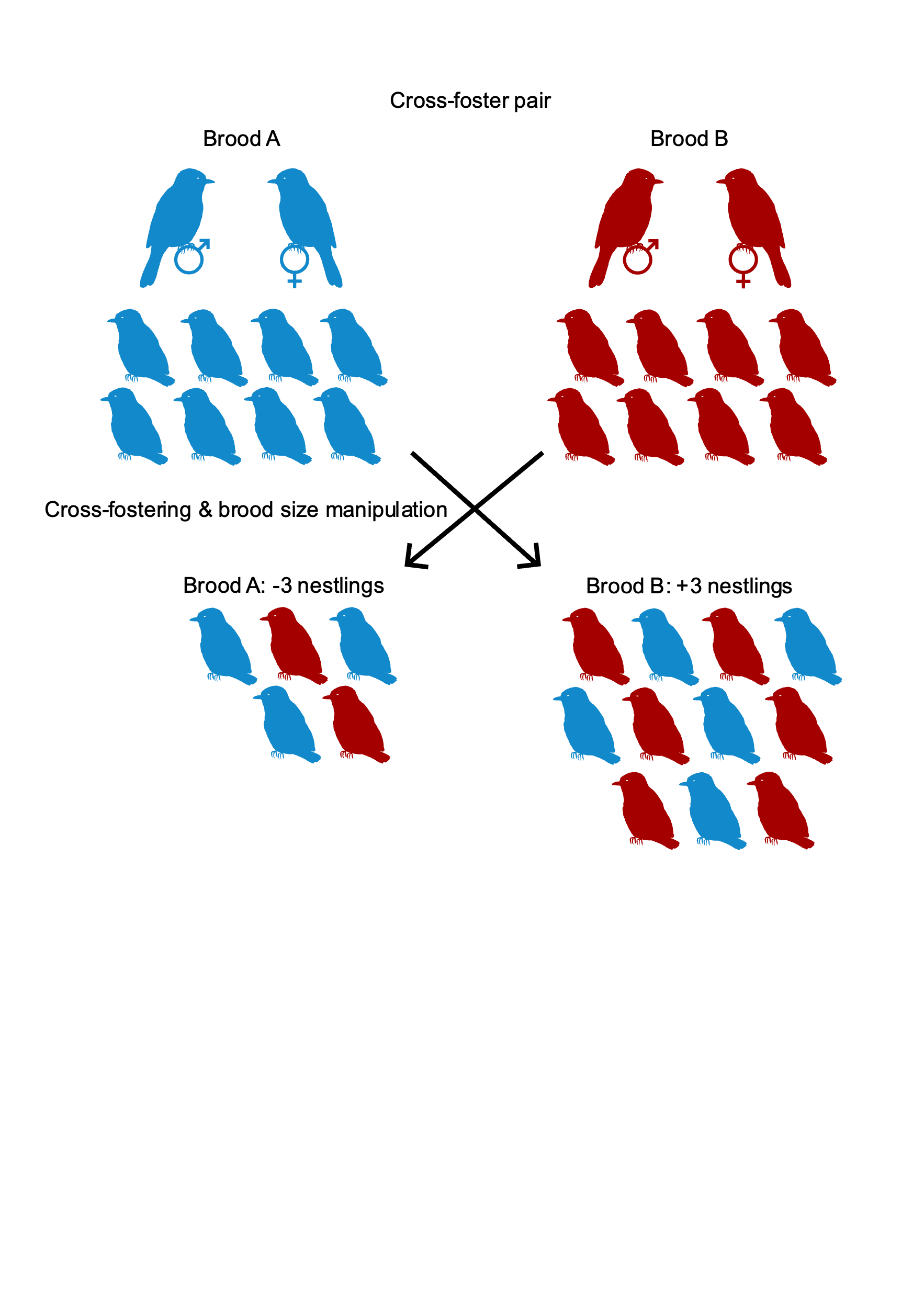


***Supplementary Figure 1.*** *Schematic overview of the cross-fostering procedure and the brood size manipulation within one cross-foster pair. The part above the arrows shows two unmanipulated broods, each consisting of eight nestlings. One brood is depicted in red and one in blue, which indicates relatedness (biological siblings and their biological parents). The part below the arrows shows the final distribution of the nestlings over the two broods after cross-fostering and brood size manipulation. Nestlings were partially swapped within the cross-foster pair and brood sizes were manipulated by enlarging the original brood size of the red brood by three nestlings while the original brood size of the blue brood was reduced by three nestlings. This procedure was performed with 16 cross-foster pairs, which resulted in 16 experimentally reduced and 16 experimentally enlarged broods.*

| ***Supplementary Table 1.*** *Overview of statistical analyses for each data type, including relevant tables and figures.* | | | | |
| --- | --- | --- | --- | --- |
| **Data type** | **Objective** | **Analysis** | **Tables** | **Figures** |
| Days since fledging | Independence hatch date | Spearman's rank-order correlation | NA | NA |
| Days since fledging | Independence date of testing | Spearman's rank-order correlation | NA | NA |
| Weight | Time since fledging - treatment effect | LMM | Suppl. Table 4 | Fig. 1a |
| ∆ weight | Time since fledging - treatment effect | LMM & post hoc with emtrends | Suppl. Table 5 | Fig. 1b & 2 |
| P3 length | Time since fledging - treatment effect | LMM & post hoc with emtrends | Suppl. Table 6 | Fig. 1c |
| Exploratory behaviour | Time since fledging - treatment effect | LMM & post hoc with emtrends | Suppl. Table 7 | Fig. 1d |
| CpG methylation | Time since fledging - treatment effect | GLMMs with and without interaction & post hoc with emtrends | Suppl. Table 3 | Fig. 3 - 5 & Suppl. Fig. 2 |
| 420 days-dependent DMS | Biological interpretation | GO analysis using Cluego and String | Suppl. Tables 8 - 14 | Suppl. Fig. 3 |

| ***Supplementary Table 2.*** *Number of CpG sites before and after filtering for the post-fledging epiGBS2 data set.* | |
| --- | --- |
| **Number of unique CpGs** | |
| **Raw** | 4,032,127 |
| **Destranding** | 2,162,591 |
| **N ≥ 15** | 1,657,280 |
| **Coverage ≥ 10x** | 1,616,679 |
| **≤ 99.9^th^ percentile of coverage** | 1,616,667 |
| **N ≥ 25 or N ≥ 15** | 281,489 |
| **0.05 ≥ mean meth ≤ 0.95** | 116,064 |

| ***Supplementary Table 3.*** *Number of CpG sites before and after running the four models and applying corrections for the epiGBS2 data set. The last row shows the number of DMS.* | | |
| --- | --- | --- |
|  | **Number of unique CpGs** | |
|  | **Treatment x**  **Days since fledging** | **Treatment** |
| **Filtering step:** |  |  |
| **Model output** | 116,064 | 116,064 |
| **Removal of warnings** | 112,165 | 112,542 |
| **≤ 95% HDI dispersion statistic** | 107,624 | 107,828 |
| **FDR q-value < 0.1** | 420 | 0 |

| ***Supplementary Table 4.*** ***Weight post-fledging minimum adequate model.*** *Table consists of all factors tested in the linear mixed model with the post-fledging weight of individuals from enlarged and reduced broods as the dependent variable. Brood of origin and brood of rearing both nested within CF pair were included as random factors. The estimate, degrees of freedom (numerator df and denominator df), the test statistic (F-value) and the significance (p-value) are given.* | | | | | | |
| --- | --- | --- | --- | --- | --- | --- |
|  | **Estimate ± SE** | **Num. df** | **Denom. df** | **F-value** | | **P-value** |
| **Weight post-fledging** | | | | | | |
| Minimal adequate model: | | | | | | |
| Intercept | 14.51 ± 0.56 | 1 | 58.55 | - | | - |
| Treatment (reduced) | -0.007 ± 0.33 | 1 | 20.15 | 0.0004 | | 0.98 |
| Days since fledging | 0.03 ± 0.006 | 1 | 52.85 | 19.69 | | **<0.001** |
| Dropped terms: | | | | | | |
| Treatment (reduced) x  Days since fledging | -0.02 ± 0.01 | 1 | 51.66 | 2.38 | | 0.13 |
| ***Supplementary Table 5.*** *∆* ***Weight post-fledging minimum adequate model.*** *Table consists of all factors tested in the linear mixed model with ∆* *weight* *of individuals from enlarged and reduced broods as the dependent variable. Brood of origin and brood of rearing both nested within CF pair were included as random factors. The estimate, degrees of freedom (numerator df and denominator df), the test statistic (F-value) and the significance (p-value) are given.* | | | | | | |
|  | **Estimate ± SE** | **Num. df** | **Denom. df** | **F-value** | **P-value** | |
| ∆ **Weight post-fledging** | | | | | | |
| Minimal adequate model: | | | | | | |
| Intercept | 29.31 ± 52.57 | 1 | 22.66 | - | - | |
| Treatment (reduced) | 5.31 ± 2.59 | 1 | 18.97 | 4.22 | 0.05 | |
| Days since fledging | 0.03 ± 0.005 | 1 | 50.02 | 39.01 | **<0.001** | |
| Hatch date | -2.07 ± 2.85 | 1 | 22.84 | 0.53 | 0.47 | |
| Hatch date^2^ | 0.03 ± 0.04 | 1 | 22.95 | 0.63 | 0.44 | |
| Treatment (reduced) × Hatch date^2^ | -0.005 ± 0.002 | 1 | 19.45 | 5.68 | **0.03** | |
| Dropped terms: | | | | | | |
| Treatment (reduced) × Days since fledging | -0.02 ± 0.01 | 1 | 47.03 | 2.45 | 0.12 | |
| Treatment (reduced) × Hatch date | 4.32 ± 5.47 | 1 | 16.29 | 0.63 | 0.44 | |

| ***Supplementary Table 6.*** ***P3 length post-fledging minimum adequate model.*** *Table consists of all factors tested in the linear mixed model with the P3 length of individuals from enlarged and reduced broods as the dependent variable. Brood of origin and brood of rearing both nested within CF pair were included as random factors. The estimate, degrees of freedom (numerator df and denominator df), the test statistic (F-value) and the significance (p-value) are given.* | | | | | |
| --- | --- | --- | --- | --- | --- |
|  | **Estimate ± SE** | **Num. df** | **Denom. df** | **F-value** | **P-value** |
| **P3 length post-fledging** | | | | | |
| Minimal adequate model: | | | | | |
| Intercept | 52.55 ± 1.02 | 1 | 45.87 | - | - |
| Treatment (reduced) | 3.07 ± 1.37 | 1 | 54.74 | 5.04 | 0.03 |
| Days since fledging | 0.04 ± 0.02 | 1 | 47.19 | 2.31 | 0.13 |
| Treatment (reduced) × Days since fledging | -0.04 ± 0.02 | 1 | 42.77 | 4.29 | **0.04** |
| Dropped terms: | | | | | |
| NA | - | - | - | - | **-** |

| ***Supplementary Table 7.*** ***Exploratory score post-fledging minimum adequate model.*** *Table consists of all factors tested in the linear mixed model with the exploratory score of individuals from enlarged and reduced broods as the dependent variable. Brood of origin and brood of rearing both nested within CF pair were included as random factors. The estimate, degrees of freedom (numerator df and denominator df), the test statistic (F-value) and the significance (p-value) are given.* | | | | | |
| --- | --- | --- | --- | --- | --- |
|  | **Estimate ± SE** | **Num. df** | **Denom. df** | **F-value** | **P-value** |
| **Exploratory score post-fledging** | | | | | |
| Minimal adequate model: | | | | | |
| Intercept | 4.58 ± 2.90 | 1 | 57.89 | - | - |
| Treatment (reduced) | 13.25 ± 3.86 | 1 | 58.61 | 11.78 | **0.001** |
| Days since fledging | 0.21 ± 0.05 | 1 | 57.18 | 11.65 | **0.001** |
| Treatment (reduced) × Days since fledging | -0.21 ± 0.06 | 1 | 57.72 | 11.24 | **0.001** |
| Dropped terms: | | | | | |
| NA | - | - | - | - | - |

| ***Supplementary Table 8.*** *Number of DMS associated per region after running the GLMMs using the epiGBS2 data set.* | | |
| --- | --- | --- |
|  | **Post-fledging** | |
|  | **Treatment x**  **Days since fledging** | **Treatment** |
| **Total** | 420 | 0 |
| **No annotation** | 84 | NA |
| **Annotated** | 336 | NA |
| **Promoter** | 67 | NA |
| **Of which TSS** | 5 | NA |
| **Gene body (exon or intron)** | 199 | NA |
| **Upstream** | 31 | NA |
| **Downstream** | 39 | NA |

***Supplementary Figure 2. Six categories corresponding to the different types of interactions between treatment (reduced or enlarged) and days since fledging in explaining variation in DNA methylation.*** *Red lines represent the patterns in the enlarged treatment, dark blue lines represent the patterns in the reduced treatment.* ***Category 1.*** *P-value of slopes in both treatments < 0.05, slope in the enlarged treatment > 0, slope in the reduced treatment < 0.* ***Category 2.*** *P-value of slopes in both treatments < 0.05, slope in the enlarged treatment < 0, slope in the reduced treatment > 0.* ***Category 3.*** *P-value of slope in the enlarged treatment < 0.05, P-value of slope in the reduced treatment ≥ 0.05, slope in the enlarged treatment > 0.* ***Category 4.*** *P-value of slope in the enlarged treatment < 0.05, P-value of slope in the reduced treatment ≥ 0.05, slope in the enlarged treatment < 0.* ***Category 5.*** *P-value of slope in the enlarged treatments ≥ 0.05, P-value of slope in the reduced treatment < 0.05, slope in the reduced treatment > 0.* ***Category 6.*** *P-value of slope in the enlarged treatment ≥ 0.05, P-value of slope in the reduced treatment < 0.05, slope in the reduced treatment < 0.*

| ***Supplementary Table 9.*** *Enriched GO terms for the ontology biological process with significant FDR q-values for all the genes associated with a days-dependent DMS. The number of genes in the target list annotated to the particular GO category and the number of genes in the background list annotated to a certain GO category are shown in the x and n columns, respectively. The total number of (recognised) genes in the target list was 234, the total number of genes in the background list was 7633.* | | | | | |
| --- | --- | --- | --- | --- | --- |
| **GO term** | **Description** | **P-value** | **FDR q-value** | **x** | **n** |
| **GO:0099601** | regulation of neurotransmitter receptor activity | 0.00 | 0.06 | 7 | 46 |
| **GO:0090092** | regulation of transmembrane receptor protein serine/threonine kinase signaling pathway | 0.02 | 0.07 | 11 | 164 |
| **GO:0045667** | regulation of osteoblast differentiation | 0.01 | 0.07 | 7 | 82 |
| **GO:0015276** | ligand-gated ion channel activity | 0.01 | 0.07 | 9 | 122 |
| **GO:0022834** | ligand-gated channel activity | 0.01 | 0.07 | 9 | 122 |
| **GO:0006022** | aminoglycan metabolic process | 0.02 | 0.07 | 7 | 87 |
| **GO:2001257** | regulation of cation channel activity | 0.02 | 0.07 | 8 | 107 |
| **GO:0005230** | extracellular ligand-gated ion channel activity | 0.02 | 0.07 | 6 | 68 |
| **GO:0060291** | long-term synaptic potentiation | 0.02 | 0.07 | 5 | 50 |
| **GO:0060627** | regulation of vesicle-mediated transport | 0.01 | 0.07 | 16 | 276 |
| **GO:0022836** | gated channel activity | 0.01 | 0.07 | 15 | 251 |
| **GO:0032412** | regulation of ion transmembrane transporter activity | 0.02 | 0.07 | 10 | 150 |
| **GO:0043583** | ear development | 0.02 | 0.07 | 10 | 150 |
| **GO:0030500** | regulation of bone mineralization | 0.01 | 0.07 | 5 | 47 |
| **GO:0045807** | positive regulation of endocytosis | 0.02 | 0.07 | 5 | 51 |
| **GO:0005261** | cation channel activity | 0.02 | 0.07 | 16 | 293 |
| **GO:0022803** | passive transmembrane transporter activity | 0.01 | 0.07 | 20 | 366 |
| **GO:0015267** | channel activity | 0.01 | 0.07 | 20 | 366 |
| **GO:0016197** | endosomal transport | 0.01 | 0.07 | 9 | 128 |
| **GO:0032409** | regulation of transporter activity | 0.02 | 0.07 | 11 | 171 |
| **GO:0016247** | channel regulator activity | 0.02 | 0.07 | 6 | 71 |
| **GO:0048167** | regulation of synaptic plasticity | 0.02 | 0.07 | 8 | 116 |
| **GO:0006906** | vesicle fusion | 0.02 | 0.07 | 5 | 53 |
| **GO:0030278** | regulation of ossification | 0.02 | 0.07 | 6 | 72 |
| **GO:0060485** | mesenchyme development | 0.03 | 0.07 | 11 | 185 |
| **GO:0048762** | mesenchymal cell differentiation | 0.03 | 0.08 | 10 | 155 |
| **GO:0110149** | regulation of biomineralization | 0.03 | 0.08 | 5 | 57 |
| **GO:0070167** | regulation of biomineral tissue development | 0.03 | 0.08 | 5 | 57 |
| **GO:0022824** | transmitter-gated ion channel activity | 0.01 | 0.08 | 6 | 62 |
| ***Supplementary Table 9 (continued).*** | | | | | |
| **GO term** | **Description** | **P-value** | **FDR q-value** | **x** | **n** |
| **GO:0022835** | transmitter-gated channel activity | 0.01 | 0.08 | 6 | 62 |
| **GO:0090174** | organelle membrane fusion | 0.03 | 0.08 | 5 | 56 |
| **GO:0005216** | ion channel activity | 0.01 | 0.08 | 19 | 347 |
| **GO:0097120** | receptor localization to synapse | 0.01 | 0.08 | 5 | 38 |
| **GO:0090100** | positive regulation of transmembrane receptor protein serine/threonine kinase signaling pathway | 0.01 | 0.08 | 7 | 74 |
| **GO:0035235** | ionotropic glutamate receptor signaling pathway | 0.00 | 0.08 | 6 | 50 |
| **GO:0022898** | regulation of transmembrane transporter activity | 0.01 | 0.09 | 11 | 158 |
| **GO:0006897** | endocytosis | 0.03 | 0.09 | 15 | 285 |
| **GO:0008083** | growth factor activity | 0.03 | 0.09 | 6 | 80 |
| **GO:0099106** | ion channel regulator activity | 0.03 | 0.09 | 6 | 81 |
| **GO:1904062** | regulation of cation transmembrane transport | 0.04 | 0.09 | 12 | 211 |
| **GO:0099094** | ligand-gated cation channel activity | 0.01 | 0.09 | 9 | 108 |
| **GO:0007041** | lysosomal transport | 0.04 | 0.09 | 5 | 61 |
| **GO:0007215** | glutamate receptor signaling pathway | 0.00 | 0.09 | 7 | 65 |
| **GO:0007034** | vacuolar transport | 0.04 | 0.09 | 6 | 83 |
| **GO:0048864** | stem cell development | 0.04 | 0.09 | 5 | 62 |
| **GO:0040013** | negative regulation of locomotion | 0.04 | 0.09 | 10 | 173 |
| **GO:0016050** | vesicle organization | 0.04 | 0.09 | 10 | 173 |
| **GO:0030203** | glycosaminoglycan metabolic process | 0.04 | 0.09 | 6 | 84 |
| **GO:0050806** | positive regulation of synaptic transmission | 0.04 | 0.09 | 6 | 84 |
| **GO:0006023** | aminoglycan biosynthetic process | 0.05 | 0.09 | 5 | 65 |
| **GO:0014033** | neural crest cell differentiation | 0.05 | 0.09 | 5 | 65 |
| **GO:0007156** | homophilic cell adhesion via plasma membrane adhesion molecules | 0.04 | 0.09 | 5 | 64 |
| **GO:0034765** | regulation of ion transmembrane transport | 0.05 | 0.09 | 14 | 272 |
| **GO:0042471** | ear morphogenesis | 0.04 | 0.09 | 6 | 85 |
| **GO:0090101** | negative regulation of transmembrane receptor protein serine/threonine kinase signaling pathway | 0.05 | 0.10 | 6 | 87 |
| **GO:0007178** | transmembrane receptor protein serine/threonine kinase signaling pathway | 0.04 | 0.10 | 12 | 223 |

| ***Supplementary Table 10.*** *Enriched GO terms for the ontology cellular component with significant FDR q-values for all the genes associated with a days-dependent DMS. The number of genes in the target list annotated to the particular GO category and the number of genes in the background list annotated to a certain GO category are shown in the x and n columns, respectively. The total number of (recognised) genes in the target list was 234, the total number of genes in the background list was 7633.* | | | | | |
| --- | --- | --- | --- | --- | --- |
| **GO term** | **Description** | **P-value** | **FDR q-value** | **x** | **n** |
| **GO:0043679** | axon terminus | 0.02 | 0.07 | 7 | 87 |
| **GO:0097447** | dendritic tree | 0.02 | 0.07 | 20 | 393 |
| **GO:0030425** | dendrite | 0.02 | 0.07 | 20 | 392 |
| **GO:0043025** | neuronal cell body | 0.02 | 0.07 | 16 | 301 |
| **GO:0098858** | actin-based cell projection | 0.01 | 0.07 | 9 | 121 |
| **GO:0030424** | axon | 0.01 | 0.07 | 21 | 401 |
| **GO:0044306** | neuron projection terminus | 0.03 | 0.08 | 7 | 97 |
| **GO:0019898** | extrinsic component of membrane | 0.01 | 0.08 | 11 | 154 |
| **GO:0030136** | clathrin-coated vesicle | 0.01 | 0.08 | 8 | 99 |
| **GO:0098984** | neuron to neuron synapse | 0.03 | 0.09 | 13 | 241 |
| **GO:0031225** | anchored component of membrane | 0.04 | 0.09 | 6 | 83 |
| **GO:0032421** | stereocilium bundle | 0.00 | 0.09 | 5 | 37 |
| **GO:0014069** | postsynaptic density | 0.04 | 0.10 | 12 | 223 |
| **GO:0019897** | extrinsic component of plasma membrane | 0.04 | 0.10 | 6 | 86 |
| **GO:0032420** | stereocilium | 0.00 | 0.10 | 5 | 33 |

| ***Supplementary Table 11.*** *Enriched GO terms for the ontology molecular function with significant FDR q-values for all the genes associated with a days-dependent DMS. The number of genes in the target list annotated to the particular GO category and the number of genes in the background list annotated to a certain GO category are shown in the x and n columns, respectively. The total number of (recognised) genes in the target list was 234, the total number of genes in the background list was 7633.* | | | | | | | | |  |
| --- | --- | --- | --- | --- | --- | --- | --- | --- | --- |
| **GO term** | | **Description** | **P-value** | **FDR q-value** | **x** | | **n** | |  |
| **GO:0030594** | | neurotransmitter receptor activity | 0.02 | 0.07 | 7 | | 94 | |  |
| **GO:0046332** | | SMAD binding | 0.02 | 0.07 | 5 | | 53 | |  |
| **GO:0030165** | | PDZ domain binding | 0.02 | 0.07 | 5 | | 53 | |  |
| **GO:0008066** | | glutamate receptor activity | 0.01 | 0.07 | 6 | | 54 | |  |
| **GO:0001540** | | amyloid-beta binding | 0.01 | 0.08 | 5 | | 44 | |  |
| **GO:0003727** | | single-stranded RNA binding | 0.01 | 0.08 | 5 | | 42 | |  |
| **GO:0019903** | | protein phosphatase binding | 0.04 | 0.09 | 6 | | 85 | |  |
| ***Supplementary Table 12.*** *Enriched GO terms for the Kyoto Encyclopedia of Genes and Genomes (KEGG) pathway with significant FDR q-values for all the genes associated with a days-dependent DMS. The number of genes in the target list annotated to the particular GO category and the number of genes in the background list annotated to a certain GO category are shown in the x and n columns, respectively. The total number of (recognised) genes in the target list was 234, the total number of genes in the background list was 7633.* | | | | | | | | | |
| **GO term** | **Description** | | **P-value** | **FDR q-value** | | **x** | | **n** | |
| **KEGG:04613** | Neutrophil extracellular trap formation | | 0.01 | 0.07 | | 5 | | 48 | |
| **KEGG:04070** | Phosphatidylinositol signaling system | | 0.03 | 0.08 | | 5 | | 56 | |
| **KEGG:05415** | Diabetic cardiomyopathy | | 0.03 | 0.09 | | 6 | | 80 | |

| ***Supplementary Table 13.*** *Enriched GO terms for the ontology biological process with significant FDR q-values for all proteins associated with a days-dependent DMS. The number of proteins in the network annotated with a particular term and the total number of proteins (in the network and in the background) annotated with this term are shown in the x and n columns, respectively. Strength is a measure of the enrichment effect (log10(observed x/expected x)). P-values are FDR corrected for multiple testing.* | | | | | |
| --- | --- | --- | --- | --- | --- |
| **GO term** | **Description** | **Strength** | **FDR q-value** | **x** | **n** |
| **GO:0032502** | Developmental process | 0.22 | 7.92 x 10^-6^ | 115 | 5841 |
| **GO:0048731** | System development | 0.25 | 1.26 x 10^-5^ | 94 | 4426 |
| **GO:0048856** | Anatomical structure development | 0.22 | 1.43 x 10^-5^ | 107 | 5402 |
| **GO:0007275** | Multicellular organism development | 0.23 | 2.02 x 10^-5^ | 101 | 5023 |
| **GO:0007399** | Nervous system development | 0.31 | 0.0003 | 58 | 2371 |
| **GO:0009653** | Anatomical structure morphogenesis | 0.31 | 0.001 | 53 | 2165 |
| **GO:0048513** | Animal organ development | 0.25 | 0.002 | 68 | 3197 |
| **GO:0032501** | Multicellular organismal process | 0.16 | 0.003 | 118 | 6933 |
| **GO:0048699** | Generation of neurons | 0.35 | 0.003 | 41 | 1551 |
| **GO:0022008** | Neurogenesis | 0.33 | 0.006 | 42 | 1657 |
| **GO:0048869** | Cellular developmental process | 0.22 | 0.006 | 74 | 3757 |
| **GO:0030154** | Cell differentiation | 0.22 | 0.007 | 73 | 3702 |
| **GO:0030182** | Neuron differentiation | 0.39 | 0.009 | 30 | 1019 |
| **GO:0048812** | Neuron projection morphogenesis | 0.51 | 0.01 | 19 | 495 |
| **GO:1900449** | Regulation of glutamate receptor signaling pathway | 0.97 | 0.02 | 7 | 63 |
| **GO:0032989** | Cellular component morphogenesis | 0.46 | 0.02 | 21 | 614 |

| ***Supplementary Table 14.*** *Enriched GO terms for the ontology cellular component with significant FDR q-values for all proteins associated with a days-dependent DMS. The number of proteins in the network annotated with a particular term and the total number of proteins (in the network and in the background) annotated with this term are shown in the x and n columns, respectively. Strength is a measure of the enrichment effect (log10(observed x/expected x)). P-values are FDR corrected for multiple testing.* | | | | | |
| --- | --- | --- | --- | --- | --- |
| **GO term** | **Description** | **Strength** | **FDR q-value** | **x** | **n** |
| **GO:0043005** | Neuron projection | 0.36 | 0.008 | 37 | 1366 |
| **GO:0042995** | Cell projection | 0.27 | 0.01 | 51 | 2287 |
| **GO:0120025** | Plasma membrane bounded cell projection | 0.26 | 0.02 | 48 | 2193 |
| **GO:0030054** | Cell junction | 0.26 | 0.04 | 45 | 2075 |
| **GO:0030425** | Dendrite | 0.44 | 0.04 | 20 | 612 |
| **GO:0036477** | Somatodendritic compartment | 0.37 | 0.04 | 24 | 852 |
| **GO:0045202** | Synapse | 0.31 | 0.04 | 33 | 1351 |


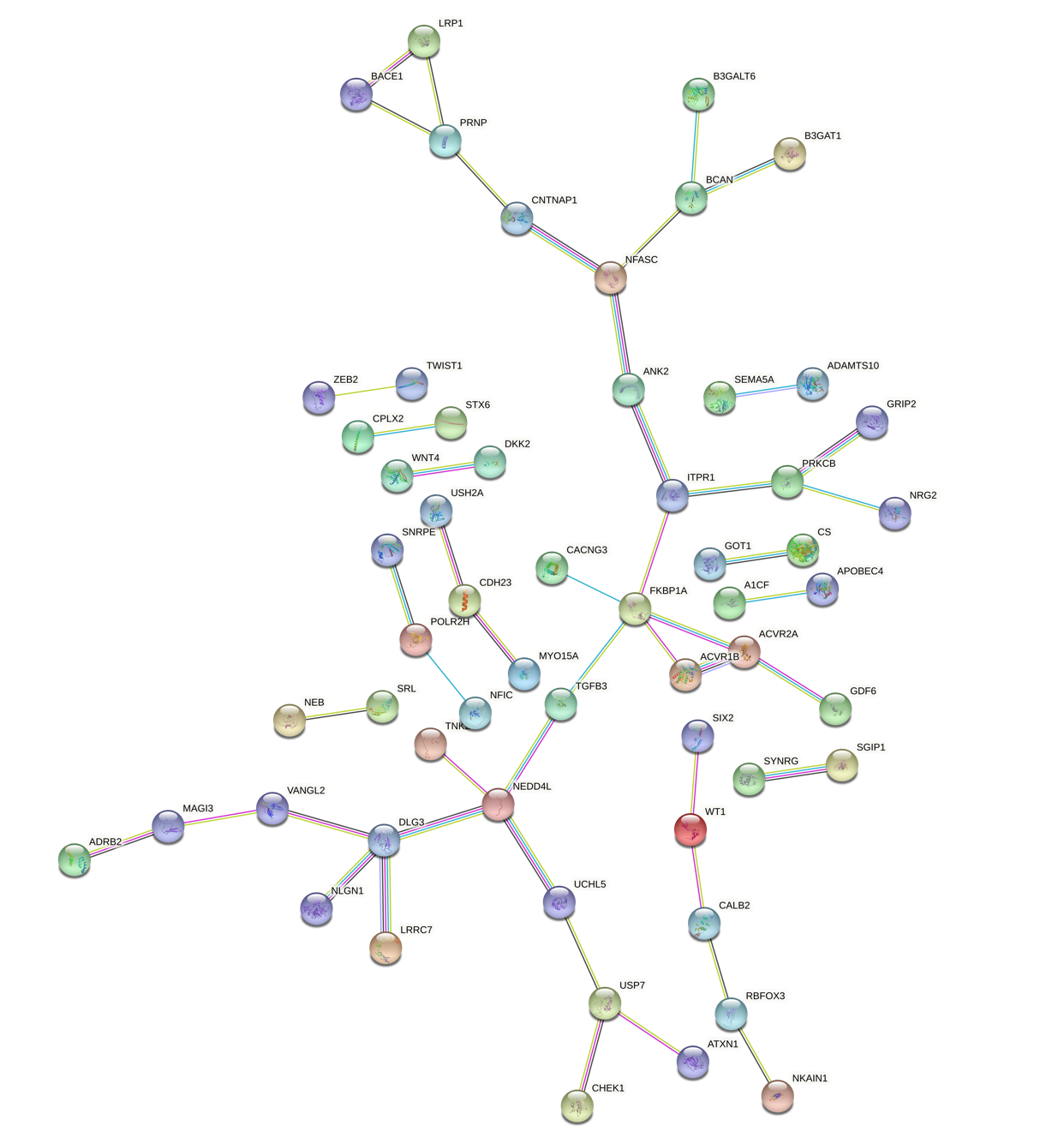


***Supplementary Figure 3****. Protein network of proteins associated with a days-dependent DMS. Joint proteins contribute to a shared function. The network was created with String.*

| ***Supplementary Table 15.*** *Days-dependent DMS in genes related to metabolism, development and growth, focusing on days-dependent DMS within regulatory regions (promoter and TSS regions) of genes and days-dependent DMS that occurred in the same gene.* | |
| --- | --- |
| **Gene** | **Findings (incl. relevant papers)** |
| *NEDD4L* | hypertension (Liang et al., 2014),  obesity (Wang et al., 2013; Keller et al., 2017) |
| *B3GALNT1* | maternal hyperglycemia (Eckmann et al., 2021),  maternal obesity (McCoski et al., 2018),  growth (Yuan et al., 2014) |
| *PHLDB2* | metabolism (see Fuselier and Lu, 2020) |
| *SKOR1* | body mass index (Schachtschneider et al., 2021) |
| *ADGRG1* | lipid deposition (Wang et al., 2017) |
| *SLC35B1*  *SLC20A2*  *SLC25A25*  *SLC25A6*  *SLC4A9* | *SLC2A8* hypomethylated in small-for-gestational-age infants experiencing catch-up growth (Diaz et al., 2020) |
| *ZNF664-like (LOC107199222)* | regulation of [glucose](https://en.wikipedia.org/wiki/Glucose) levels and [fatty acid](https://en.wikipedia.org/wiki/Fatty_acid) breakdown (Wu et al., 2014)  BMI and type 2 diabetes (Zhang et al., 2017)  Differentially methylated between great tit nestlings from enlarged and reduced broods (Sepers et al., 2021). |

| ***Supplementary Table 16.*** *Days-dependent DMS in genes related to behaviour and cognition, focusing on days-dependent DMS within regulatory regions (promoter and TSS regions) of genes and days-dependent DMS that occurred in the same gene.* | |
| --- | --- |
| **Gene** | **Findings (incl. Relevant papers)** |
| *NPD* | expression affected by diet (Nyunt et al., 2019) |
| *BSDC1* | vocal learning (Hilliard et al., 2012) |
| *SIRPB1-like (LOC107213130)* | Alzheimer’s disease (Gaikwad et al., 2009)  impulsive-disinhibited personality (Laplana et al., 2014)  human migration (Royo et al., 2018) |
| *SIRPB1-like* | depression (Yamawaki et al., 2018) |
| *NEDD4L* | depression (Xu et al., 2020) |
| *RNF24* | depression (Li et al., 2016) |
| *ADGR1* | depression (Belzeaux et al., 2020) |
| *TPH2* | biosynthesis of serotonin |
| *SLC35B1*  *SLC20A2*  *SLC25A25*  *SLC25A6*  *SLC4A9* | polymorphisms and DNA methylation in *SLC6A4* (*SERT*), which protein transports serotonin, have been linked to adverse early life conditions and stress responses (see Non et al., 2016; Parade et al., 2021). |

***Reference list Supplementary Tables 15 and 16***

Belzeaux, R., Gorgievski, V., Fiori, L. M., Lopez, J. P., Grenier, J., Lin, R., et al. (2020). GPR56/ADGRG1 is associated with response to antidepressant treatment. Nat Commun 11, 1635. doi: 10.1038/s41467-020-15423-5.

Diaz, M., Garde, E., Lopez-Bermejo, A., de Zegher, F., and Ibañez, L. (2020). Differential DNA methylation profile in infants born small-for-gestational-age: association with markers of adiposity and insulin resistance from birth to age 24 months. BMJ Open Diab Res Care 8, e001402. doi: 10.1136/bmjdrc-2020-001402.

Eckmann, M., Sheng, Q., Baldwin H, S., and Lister, R. L. (2021). Maternal Hyperglycemia Induces Changes in Gene Expression and Morphology in Mouse Placentas. Gynecol Reprod Health 5, 10.33425/2639-9342.1140. doi: 10.33425/2639-9342.1140.

Fuselier, T. T., and Lu, H. (2020). PHLD Class Proteins: A Family of New Players in the p53 Network. Int J Mol Sci 21, 3543. doi: 10.3390/ijms21103543.

Gaikwad, S., Larionov, S., Wang, Y., Dannenberg, H., Matozaki, T., Monsonego, A., et al. (2009). Signal Regulatory Protein-β1: A Microglial Modulator of Phagocytosis in Alzheimer’s Disease. The American Journal of Pathology 175, 2528–2539. doi: 10.2353/ajpath.2009.090147.

Hilliard, A. T., Miller, J. E., Fraley, E. R., Horvath, S., and White, S. A. (2012). Molecular Microcircuitry Underlies Functional Specification in a Basal Ganglia Circuit Dedicated to Vocal Learning. Neuron 73, 537–552. doi: 10.1016/j.neuron.2012.01.005.

Keller, M., Hopp, L., Liu, X., Wohland, T., Rohde, K., Cancello, R., et al. (2017). Genome-wide DNA promoter methylation and transcriptome analysis in human adipose tissue unravels novel candidate genes for obesity. Molecular Metabolism 6, 86–100. doi: 10.1016/j.molmet.2016.11.003.

Laplana, M., Royo, J. L., García, L. F., Aluja, A., Gomez-Skarmeta, J. L., and Fibla, J. (2014). SIRPB1 copy-number polymorphism as candidate quantitative trait locus for impulsive-disinhibited personality. Genes, Brain and Behavior 13, 653–662. doi: 10.1111/gbb.12154.

Li, Q. S., Tian, C., Seabrook, G. R., Drevets, W. C., and Narayan, V. A. (2016). Analysis of 23andMe antidepressant efficacy survey data: implication of circadian rhythm and neuroplasticity in bupropion response. Transl Psychiatry 6, e889. doi: 10.1038/tp.2016.171.

Liang, H., Wu, X., Chen, X., Wang, Y., Li, Y., Pan, B., et al. (2014). Gender difference in association of NEDD4L gene variants among southern Han Chinese with essential hypertension – a population-based case–control study. Clinical and Experimental Hypertension 36, 309–314. doi: 10.3109/10641963.2013.827693.

McCoski, S. R., Vailes, M. T., Owens, C. E., Cockrum, R. R., and Ealy, A. D. (2018). Exposure to maternal obesity alters gene expression in the preimplantation ovine conceptus. BMC Genomics 19, 737. doi: 10.1186/s12864-018-5120-0.

Non, A. L., Hollister, B. M., Humphreys, K. L., Childebayeva, A., Esteves, K., Zeanah, C. H., et al. (2016). DNA methylation at stress-related genes is associated with exposure to early life institutionalization. American Journal of Physical Anthropology 161, 84–93. doi: 10.1002/ajpa.23010.

Nyunt, T., Britton, M., Wanichthanarak, K., Budamagunta, M., Voss, J. C., Wilson, D. W., et al. (2019). Mitochondrial oxidative stress-induced transcript variants of ATF3 mediate lipotoxic brain microvascular injury. Free Radical Biology and Medicine 143, 25–46. doi: 10.1016/j.freeradbiomed.2019.07.024.

Parade, S. H., Huffhines, L., Daniels, T. E., Stroud, L. R., Nugent, N. R., and Tyrka, A. R. (2021). A systematic review of childhood maltreatment and DNA methylation: candidate gene and epigenome-wide approaches. Translational Psychiatry 11, 1–33. doi: 10.1038/s41398-021-01207-y.

Royo, J. L., Valls, J., Acemel, R. D., Gómez-Marin, C., Pascual-Pons, M., Lupiañez, A., et al. (2018). A common copy-number variant within SIRPB1 correlates with human Out-of-Africa migration after genetic drift correction. PLOS ONE 13, e0193614. doi: 10.1371/journal.pone.0193614.

Schachtschneider, K. M., Schook, L. B., Meudt, J. J., Shanmuganayagam, D., Zoller, J. A., Haghani, A., et al. (2021). Epigenetic clock and DNA methylation analysis of porcine models of aging and obesity. GeroScience 43, 2467–2483. doi: 10.1007/s11357-021-00439-6.

Sepers, B., Erven, J. A. M., Gawehns, F., Laine, V. N., and van Oers, K. (2021). Epigenetics and Early Life Stress: Experimental Brood Size Affects DNA Methylation in Great Tits (Parus major). Frontiers in Ecology and Evolution 9. doi: 10.3389/fevo.2021.609061.

Wang, L., Li, X., Ma, J., Zhang, Y., and Zhang, H. (2017). Integrating genome and transcriptome profiling for elucidating the mechanism of muscle growth and lipid deposition in Pekin ducks. Sci Rep 7, 3837. doi: 10.1038/s41598-017-04178-7.

Wang, Y. L., Liang, H. Y., Gao, Y. H., Wu, X. J., Chen, X., Pan, B. Y., et al. (2013). A Functional Variant of NEDD4L Is Associated with Obesity and Related Phenotypes in a Han Population of Southern China. International Journal of Molecular Sciences 14, 7433–7444. doi: 10.3390/ijms14047433.

Xu, J., Guo, C., Liu, Y., Wu, G., Ke, D., Wang, Q., et al. (2020). Nedd4l downregulation of NRG1 in the mPFC induces depression-like behaviour in CSDS mice. Transl Psychiatry 10, 249. doi: 10.1038/s41398-020-00935-x.

Yamawaki, Y., Yoshioka, N., Nozaki, K., Ito, H., Oda, K., Harada, K., et al. (2018). Sodium butyrate abolishes lipopolysaccharide-induced depression-like behaviors and hippocampal microglial activation in mice. Brain Research 1680, 13–38. doi: 10.1016/j.brainres.2017.12.004.

Yuan, Z., Song, D., and Wang, Y. (2014). The novel gene pFAM134B positively regulates fat deposition in the subcutaneous fat of Sus scrofa. Biochemical and Biophysical Research Communications 454, 554–559. doi: 10.1016/j.bbrc.2014.10.117.
